# Principles for Strengthening the Integrity of Clinical Research 

**DOI:** 10.1371/journal.pctr.0010001

**Published:** 2006-04-21

**Authors:** David Korn, Susan Ehringhaus

“All true universities, whether public or private, are public trusts designed to advance knowledge by safeguarding the free inquiry of impartial teachers and scholars. Their independence is essential because the university provides knowledge not only to its students, but also to the public agency in need of expert guidance and the general society in need of greater knowledge;... these latter clients have a stake in disinterested professional opinion, stated without fear or favor, which the institution is morally required to respect.” — American Association of University Professors [1]

Public skepticism about the timeliness, accuracy, and completeness of reporting clinical trial results has never been more pervasive. The topic continues to attract attention from the media and the United States Congress [2,3]. Recent allegations that have shaken public confidence include suppression of studies of antidepressants in adolescents that failed to show effectiveness [4] and failure to describe adequately the cardiovascular risks of some COX-2 inhibitors, most notably Vioxx (rofecoxib) [5]. Because such clinical trials often involve participation by medical schools, teaching hospitals, and prominent faculty frequently sought after by industry as “thought leaders,” this climate of unease and mistrust severely challenges the integrity of academic medicine as well as that of biopharmaceutical sponsors.

Some sectors of the international clinical trials enterprise have begun to respond to the gathering crisis of confidence. The editors of major medical journals, particularly the members of the International Committee of Medical Journal Editors (ICMJE), have promulgated strong positions on authorship [6] and mandatory “full” registration of clinical trials in publicly funded, freely accessible registries [7,8]. The National Institutes of Health (NIH) has established the sole publicly funded and operated clinical trials registry in the United States, accessible at http://www.clinicaltrials.gov [9]. The World Health Organization (WHO) has defined a “minimum dataset” for clinical trials registration and continues to negotiate the details of its universal adoption by industry [10,11]. Comprehensive legislation (the Fair Access to Clinical Trials Act of 2005 [12] and the American Center for Cures Act of 2005 [13]) has been introduced in the United States Congress. The Public Library of Science (PLoS) has partnered with the American Medical Informatics Association to develop the Global Trial Bank [14], the first independently operated, peer-reviewed, freely accessible repository for clinical trial results. PLoS' newest open-access journal, *PLoS Clinical Trials,* is committed to publishing the results of ethically and scientifically sound clinical trials without regard to their direction or perceived importance [15]. These developments are encouraging, notwithstanding a continued absence of consensus about the specific identifying information that should be registered for each clinical trial, the format and detail with which trial results should be deposited, and how much of, and at what point, this information should be publicly accessible [16].

Not so encouraging is the significant variation across the academic community in standards that protect the right and duty of academic investigators to take appropriate responsibility for the design, analysis, and reporting of clinical research, especially clinical trials sponsored by industry [17,18]. Such variation is troubling for many reasons, but two are of cardinal importance. First, clinical research involving human participants can only be justified ethically when such experiments are done to produce generalizable knowledge [19,20]. We and others [21] interpret that dictum to mean that the results of human experimentation should be made known. Second, academic medical institutions and faculty have occupied an especially privileged place in society as stewards and trustworthy sources of the independent and impartial research, accurate information, and unbiased interpretation that are necessary for society to make sound policy choices.

Because inconsistency in research standards can affront human research ethics, undermine academic integrity, distort public policy and medical practice, and impair public health, the Association of American Medical Colleges (AAMC), in collaboration with the Centers for Education and Research in Therapeutics and the BlueCross BlueShield Association, convened in June 2005 a panel of nationally recognized experts and developed a set of principles for conducting and reporting clinical research. (See Box 1.) The principles were endorsed by AAMC's governance in September 2005 and have been shared widely with medical, scientific, and patient organizations and with senior biopharmaceutical executives, with the goal to identify areas of agreement and concern among these diverse stakeholders in clinical research. In response to these comments, the document was revised to resolve ambiguous language and clarify certain technical requirements, and the final version appeared in January 2006 [22].

The principles express the fundamental responsibilities of academic institutions and faculty for research conducted under their auspices. Accordingly, they should apply to all clinical trials conducted in academic medical institutions, regardless of the source of funding. They encompass single-site as well as multisite studies, although the actual application of the principles may differ in detail across study types and sizes. “Clinical trials” are defined here in accordance with the ICMJE standards that explicitly exclude Phase 1 and early (exploratory) Phase 2 studies (but not all late-Phase 2 studies) and that include all Phase 3 and 4 clinical trials, including studies of new indications for approved products [7,8].

Whenever principles of engagement are promulgated, the challenge is to win the allegiance of those whose interests are most at stake. Although the principles presented here emanate from fundamental tenets of sound scientific scholarship and human research ethics—and should be applicable, to the extent appropriate, to all clinical research involving human participants, regardless of site or sponsorship—they will doubtless challenge both academia and industry. For academic institutions, the rapid doubling of the NIH budget spurred major increases in financial investments and indebtedness and expanded biomedical research capacity. The recent abrupt flattening of NIH appropriations [23] makes commercial funding ever more attractive to researchers and may tempt academic institutions to stretch—or ignore—their policies to appear more accommodating to industry sponsors.

**Box 1. Principles for Protecting Integrity in the Conduct and Reporting of Clinical Trials**

These appear verbatim from [22].

**Publications and Public Availability of Research Results**

1. Researchers and their institutions have an ethical obligation when conducting human research to seek to make the results available publicly.

2. Contracts between sponsors and institutions for conducting clinical trials should require a good faith effort to publish the results of such trials in a peer reviewed journal in a timely fashion.

3. Contracts for clinical trials should contain a commitment of adequate funding to cover the full costs of the analysis defined in the protocol and the costs associated with publishing the results. This principle applies even when the study is terminated for any reason prior to meeting its pre-specified objectives.

4. All trials meeting the ICMJE requirements [7,8] for registration should make their results publicly available, by means of a link to any peer reviewed publications and by posting the results in an online accessible repository, within 18 months of submission of a manuscript for publication. (The WHO is leading an international effort to promote registration of clinical trials, but has not yet gained consensus on the issue of “masking” of certain elements in the minimum data set required for registration. Because of continuing uncertainty, the WHO effort is acknowledged but not included as an alternative to the ICMJE registration requirements.)

5. After publication of the results, the sponsor, the investigators, and their institutions should adopt a model for public sharing of the data underlying publications, similar to that of NIH [28], which permits exceptions for confidential or proprietary information.

**Registration of Clinical Trials**

6. Within 21 days of initiating enrollment of participants, any clinical trial covered by these principles should be fully registered pursuant to the ICMJE requirements [7,8] for registration. Registration must include the assignment of a unique identifying number to each clinical trial.

7. Registration should be accomplished either in clinicaltrials.gov or in another public, non-profit, international registry and should include all of the elements required by that registry.

8. Insofar as is feasible, trial registration data should be regularly updated to include a link to all published reports associated with the study.

**Lead Investigator and Steering Committee**

9. A multisite clinical trial, at the outset, should identify a lead or principal investigator and a steering committee to represent the full body of investigators.

**Publication and Analysis Committee**

10. A multisite clinical trial, at the outset, should establish a publication and analysis committee [hereinafter P&A committee]. It is essential that the P&A committee be independent of the sponsor's control, have access to the full data set, understand and implement the prespecified analysis plan, and have the resources and skills both to interpret that analysis and perform additional analysis if required. In order to prevent any appearance of undue influence by the sponsor, the P&A committee should contain a majority of participating, non-sponsor-employed investigators, with appropriate skills in analysis and interpretation of clinical trials. The P&A committee and the steering committee may have the same membership.

11. The P&A committee in multisite clinical trials (or the principal investigator of single site studies), through a qualified expert of its choosing, preferably a member of that committee, should have the right to access any data generated during the study that the committee deems necessary to ensure the integrity and validity of the study and its full reporting.

12. The P&A committee in multisite clinical trials (or the principal investigator in single site studies) should require that the sponsor of the study perform its analysis of trial data in a defined period of time. The committee (or PI) should be able to conduct its own analysis through an expert selected by it, to the extent it deems this necessary. Whenever feasible, the expert should be agreed upon by the P&A committee and the sponsor.

13. The sponsor should share with the P&A committee all analyses called for by the study that the sponsor conducts of any biological materials it receives during the course of the study.

14. The P&A committee or PI should make a good faith effort to disseminate the results of the study through peer reviewed mechanisms.

**Individual Publication**

15. Site-specific publications in multisite trials have an unavoidable potential for bias. Because they are almost never part of the original analytic plan, they are often misleading, and should be strongly discouraged. However, to respect an academic institution's commitment to academic freedom, site-specific analyses should nonetheless be permitted with conditions. Accordingly, an individual site investigator in a multisite trial should be free to analyze and publish data from the individual site, consistent with sound principles of science and analysis, but *only* after review and comment by the P&A committee and *only* after publication of the study as a whole, or, in the absence of acceptance of the full publication, within 2 years from the specified end points or earlier termination of the study.

**Authorship**

16. Ghost or guest authorship is unacceptable. Authorship implies independent, substantial, and fully disclosed participation in the study and in the preparation of the manuscript. It is acceptable for employees of the sponsor to participate in drafting and publication activity, but only if their roles are fully disclosed.

17. Institutions conducting clinical trials should adopt as policy the standards of authorship defined by the ICMJE.

18. Where applicable, investigators should use the CONSORT principles [29,30] as guidance for publication of trial results.

19. Investigators should fully disclose, and journals should publish, the existence of all relevant financial interests, including consultancies of any investigator, in all communications of trial results.

20. Any manuscript submitted for publication should accurately disclose the role of each author in conducting the study and preparing the manuscript. Such information should also be disclosed in any public presentation of study results, to the extent practicable.

21. Manuscripts submitted for publication should disclose all previous publications involving the same protocol or database.

22. Manuscripts submitted for publication should be accompanied by the protocol and pre-specified analysis plan and all dated amendments to them, and any deviations to the pre-specified plan should be identified and discussed.

**Key Messages**

- There is growing public skepticism about the accuracy and completeness of reporting of clinical trial results.
- Academic medical institutions and their faculty often play leading roles in performing and reporting clinical trials.
- Clinical research in humans can only be justified ethically when it leads to generalizable knowledge, which means results should be disclosed.
- AAMC has developed principles for conducting and reporting clinical research that ensure to investigators full right of independent access to and analysis of the underlying data.
- The principles aim to ensure that the conduct and reporting of clinical research conform to the highest standards of scientific and ethical integrity.

For the biopharmaceutical industry, widely publicized concerns about “dry pipelines” [24], expiring patents that threaten operating margins, questionable practices in clinical trials, and plunging public esteem [25,26] may only intensify the industry's long-standing frustration and impatience with academic bureaucracy, especially in negotiating clinical trial agreements and navigating multiplicative institutional review board evaluations, while other options like private, for-profit clinical trial sites and cadres of willing community physicians beckon.

For academic investigators eager to participate in industry-sponsored trials to help create new medical knowledge and advance their own professional development and for their healthcare institutions that find hosting such trials important marketing assets, the universities' efforts to protect academic freedom often lead to protracted negotiations over the fine language of clinical trials contracts, and the process breeds discouragement, cynicism, and frustration in all parties. And, of course, the patients who might wish to participate in the trials are the ultimate victims of these clashing institutional interests.

Notwithstanding these formidable impediments, we are optimistic that the principles outlined here will be adopted widely across the professional community as expressions of long-endorsed values and practices. We also hope they will be championed by patients, potential research participants, and their advocacy groups, for whom this effort to strengthen the integrity and credibility of clinical trials results and the evidentiary base of medical practice can only be beneficial.

Both industry and academia have been punished in the last decade by harsh publicity and congressional rebukes over revelations of questionable (or worse) practices in conducting and reporting clinical research, hidden financial conflicts of interest, and tragic clinical research mishaps. Both sectors may welcome the opportunity to reaffirm more confidently that their partnership in clinical research, so vital for translating scientific advancements into better health care and improved public health, is “principled, protective of research subjects, and capable of withstanding intense public scrutiny” [27]. We argue that the adoption and consistent practice of these principles will contribute greatly to that outcome.

## Abbreviations

AAMC: Association of American Medical Colleges
ICMJE: International Committee of Medical Journal Editors
NIH: National Institutes of Health
P&A committee: publication and analysis committee
WHO: World Health Organization
